# Validating online approaches for rare disease research using latent class mixture modeling

**DOI:** 10.1186/s13023-021-01827-z

**Published:** 2021-05-10

**Authors:** Andrew A. Dwyer, Ziwei Zeng, Christopher S. Lee

**Affiliations:** 1 Boston College Connell School of Nursing, Chestnut Hill, MA USA; 2 Massachusetts General Hospital – Harvard Center for Reproductive Medicine, Boston, MA USA; 3 Boston College Lynch School of Education and Human Development, Center for Measurement, Evaluation, Statistics and Assessment, Chestnut Hill, MA USA; 4 Eileen O’Connor Institute of Nursing Research, Australian Catholic University, Melbourne, Australia

**Keywords:** Community based participatory research, Diagnostic odyssey, Hypogonadotropic hypogonadism, Kallmann syndrome, Patient organization, Rare disease

## Abstract

**Background:**

Rare disease patients are geographically dispersed, posing challenges to research. Some researchers have partnered with patient organizations and used web-based approaches to overcome geographic recruitment barriers. Critics of such methods claim that samples are homogenous and do not represent the broader patient population—as patients recruited from patient organizations are thought to have high levels of needs. We applied latent class mixture modeling (LCMM) to define patient clusters based on underlying characteristics. We used previously collected data from a cohort of patients with congenital hypogonadotropic hypogonadism who were recruited online in collaboration with a patient organization. Patient demographics, clinical information, Revised Illness Perception Questionnaire (IPQ-R) scores and Zung self-rating depression Scale (SDS) were used as variables for LCMM analysis. Specifically, we aimed to test the classic critique that patients recruited online in collaboration with a patient organization are a homogenous group with high needs. We hypothesized that distinct classes (clinical profiles) of patients could be identified—thereby demonstrating the validity of online recruitment and supporting transferability of findings.

**Results:**

In total, 154 patients with CHH were included. The LCMM analysis identified three distinct subgroups (Class I: n = 84 [54.5%], Class II: n = 41 [26.6%], Class III: n = 29 [18.8%]) that differed significantly in terms of age, education, disease consequences, emotional consequences, illness coherence and depression symptoms (all *p* < 0.001) as well as age at diagnosis (*p* = 0.045). Classes depict a continuum of psychosocial impact ranging from severe to relatively modest. Additional analyses revealed later diagnosis (Class I: 19.2 ± 6.7 years [95% CI 17.8–20.7]) is significantly associated with worse psychological adaptation and coping as assessed by disease consequences, emotional responses, making sense of one’s illness and SDS depressive symptoms (all *p* < 0.001).

**Conclusions:**

We identify three distinct classes of patients who were recruited online in collaboration with a patient organization. Findings refute prior critiques of patient partnership and web-based recruitment for rare disease research. This is the first empirical data suggesting negative psychosocial sequelae of later diagnosis (“diagnostic odyssey”) often observed in CHH.

## Background

Patients with rare diseases are dispersed geographically posing significant challenges to research in rare diseases [1, 2]. As such, many rare disease publications report relatively small sample sizes and/or cohorts amassed at individual centers. While geographic distance hampers prospective studies [3], it also contributes to the sense of isolation and marginalization experienced by rare disease patients [4]. The internet has been a powerful tool for rare disease patients to find information, obtain peer-to-peer support and locate online patient organizations [5]. Researchers also have leveraged the internet to enhance prospective recruitment of rare disease patients [6–8]. Additionally, researchers have collaborated with patient advocacy groups (i.e. support organizations) to enhance recruitment [8–10]. Further, some investigators have shifted from traditional transactional research paradigms to one that accepts patients as partners and key stakeholders (i.e. community-based participatory methods) [11]. One example of using community engagement and online methods comes from research on congenital hypogonadotropic hypogonadism (CHH).

Congenital hypogonadotropic hypogonadism (CHH, ORPHA174590) is a rare, genetic endocrine disorder characterized by absent/incomplete puberty and infertility resulting from deficient secretion (or action) of gonadotropin releasing hormone (GnRH). A range of non-reproductive phenotypes are associated with CHH (i.e. midline defects, skeletal/dental anomalies, unilateral renal agenesis, synkinesia/mirror movement), and approximately half of patients exhibit diminished/absent sense of smell (anosmia)—termed Kallmann syndrome (KS, ORPHA478) [12]. Incidence of CHH is estimated to be 1:48,000 [13] with a striking sexual discordance (4 males:1 female) [14]. Unlike many rare diseases, effective treatments are available. Hormonal therapies (i.e. sex steroids) induce secondary sexual characteristics and gonadotropin therapy or pulsatile GnRH can induce fertility in approximately 75–80% of cases [12]. While CHH/KS does not shorten life expectancy, there is evidence of life altering effects and significant impact on wellbeing and health-related quality of life [15, 16]. To reach geographically dispersed patients with CHH/KS, we have previously partnered with a patient organization and used online data collection to conduct patient needs assessments [17]. After identifying unmet needs, we collaborated with patients to co-create education materials responding to unmet patient informational needs, then evaluated the materials online [18, 19]. A common criticism of such participatory projects is that recruiting patients online in collaboration with a patient advocacy group creates a biased sample that is not representative of the broader patient community [20, 21]. Specifically, critics posit that identifying patients through a patient organization skews the sample—as these individuals are thought to be a homogenous group with disproportionately higher levels of need [22].

In this study we apply a novel statistical approach (latent class mixture modeling, LCMM) [23] to analyze an existing rare disease (CHH/KS) data set previously obtained by partnering with a patient organization using online data collection. We aimed to determine if distinct subgroups (classes) of patients could be identified based on demographic, clinical and patient-reported outcome data. Identifying multiple classes would refute the critique that patients recruited over the web via patient organizations are homogenous (a single class) and not representative of the larger patient population. Notably, demonstrating multiple subgroups in the CHH/KS cohort would support the validity of such recruitment approaches and bolster evidence of transferability of findings. Moreover, such evidence could strengthen methodologic rigor for internet recruitment conducted in collaboration with patient organizations, and therefore has implications for the broader rare disease research community.

## Methods

The study is a secondary analysis of de-identified quantitative data previously collected as part of a cross-sectional, multiple methods (quantitative and qualitative) needs assessment of patients with CHH/KS. The original study received ethics approval and all participants provided opt-in electronic informed consent prior to competing an online survey. Findings of the needs assessments have been previously reported [14, 17, 24, 25].

### Participants

The original study utilized a community based participatory research framework [26, 27]. Briefly, we partnered with CHH/KS patient community leaders to develop survey content, beta test the online survey and aid in recruitment (for details see [17]). Participants were recruited for the quantitative online survey via social media and patient-oriented sites (i.e. Facebook, Rareconnect.org), an online patient-led forum (CHH/KS chat room) as well as postings on www.clinicaltrials.gov and www.gnrhdeficiency.eu (COST Action BM1105). Men and women with CHH/KS [28] (18–70 years old) were included in the study. Diagnosis was confirmed in a random sampling (40% of subjects) to ensure accuracy of self-reported diagnosis.

### Instruments

The online survey collected demographic information (e.g. age, education), clinical information (i.e. age at diagnosis, seen at specialized academic medical center) and participants completed several validated instruments. The Illness Perception Questionnaire-Revised (IPQ-R) includes 38 items (scored on 5-point a Likert scale) assessing emotional and cognitive representations of illness [29]. For the present study we utilized composite scores on three dimensions: consequences (i.e. negative consequences of the disease, 6 items, range 6–30, higher scores indicate greater negative disease consequences), emotional representations (i.e. emotional responses generated by the illness, 6 items, range 6–30, higher scores indicate greater emotional impact of the disease), and illness coherence (i.e. personal understanding and making sense of the disease, 5 items, range 5–25, higher scores indicate greater understanding of the disease) [29]. The Zung Self-Rating Depression Scale (SDS) is a validated, 20-item instrument that is used widely to quantify the severity of affective, somatic, psychomotor, and psychological depressive symptoms [30, 31]. Scores range from 20 to 80 with higher scores indicating more severe depressive symptoms. A score of < 50 is considered normal, 51–60 mild clinically meaningful depressive symptoms, 61–70 moderate to major depressive symptoms (e.g. dysthymia) and > 70 akin to severe major depressive symptoms [30, 31]. For the present study we used the composite SDS score.

### Statistical analyses

**Fig. 1:**
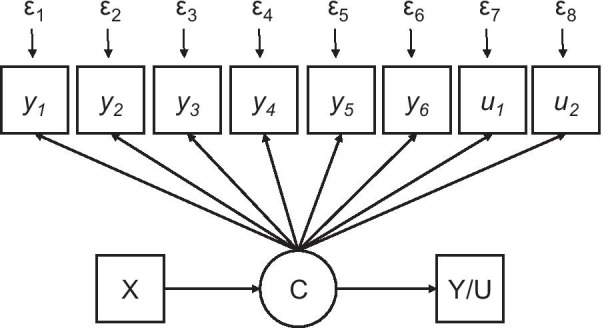
Schematic of latent class mixture modeling for the CHH/KS cohort (n = 154). The latent categorical variable (i.e. distinct class) is measured by eight (*y*_1–6_, *u*_1–2_). Continuous variables are depicted by “*y*”, binary/categorical variables “*u*” and “ε” indicates error. The categorical variable “C” indicates the most likely class for each case based on conditional probabilities. Class membership can be modeled as a function of multiple characteristics (X). Class membership can be used to predict continuous and categorical (Y/U) variables

We employed latent class mixture modeling (LCMM) to test our hypothesis that multiple distinct subgroups (classes) of patients could be identified within a rare disease cohort (Fig. 1). Briefly, LCMM is a versatile analytic strategy used to identify previously-unobserved subgroups (i.e. classes) in cross-sectional data [23]. LCMM utilizes multiple measured variables (continuous or binary/categorical) to identify subgroups of latent (unmeasured) constructs. Mixture refers to the presence of multiple subgroups (classes) with unique characteristics within a sample. LCMM produces a novel categorical variable, the hitherto unobserved classes, wherein subjects are assigned to a specific class based on the statistically greatest likelihood of belonging to the particular subgroup. Findings can be used to identify membership in a respective class as well as identify what variable(s) predict class membership. Given the sample size (n = 154), we identified eight variable for LCMM analysis (i.e. n = 20 subjects per variable). We followed step-by-step procedures for LCMM to identify subgroups (classes) as described by Ram and Grimm [32]. Mplus software [33] was employed for LCMM analyses and SPSS Version 25 (IBM) was used for other statistical analyses (i.e. χ^2^, ANOVA, Sheffe post hoc tests, multiple linear regression). As an exploratory step, we used multivariate linear regression, with age at diagnosis as the dependent variable, to assess the relationship between age at diagnosis and patient-reported outcome measures. Results are reported as mean ± standard deviation and *p* values < 0.05 were considered statistically significant.

## Results

In total, 154 participants (99 males, 55 females) were included in the analysis. Characteristics of the participants as shown in Table 1. To evaluate the null hypothesis (i.e. patients are a single, homogenous, monolithic group) we tested an initial model with two unobserved subgroups compared to a single class model followed by sequential increases in classes (i.e. 3 then four) to identify the best fit model. Based on the Ram criteria in general (i.e. Vuong–Lo–Mendell–Rubin likelihood ratio test, Lo–Mendell–Rubin adjusted likelihood ratio test), Bayesian information criteria (BIC = 6656.46) and parametric bootstrapped likelihood ratio test (*p* = 0.03) in particular, the three class solution provided more information compared with two and four class solutions. Increasing the number of classes to four failed to reach statistical significance and was abandoned in favor of the model with three subgroups.

**Table 1:** Participant characteristics (n = 154)

	Males (n = 99)	Females (n = 55)	Total (n = 154)
**Sociodemographics**			
Age (years) Mean ± SD (range)	36.8 ± 10.8 (19–65)	35.2 ± 9.7 (18–68)	36.2 ± 10.5 (18–68)
Education (n, %)			
High school	33 (33%)	10 (18%)	43 (28%)
University	35 (35%)	16 (29%)	51 (33%)
Post-graduate	31 (31%)	29 (53%)	60 (39%)
Not reported	0	1 (< 1%)	1 (< 1%)
Relationship status (n, %)			
Never been in a relationship	23 (23%)	4 (7%)	27 (18%)
Single	24 (24%)	9 (16%)	33 (21%)
Dating/in a relationship	15 (15%)	14 (25%)	29 (19%)
Married	36 (36%)	21 (38%)	57 (37%)
Divorced	1 (1%)	7 (13%)	8 (5%)
**Clinical information**			
Age at diagnosis (years) Mean ± SD (range)	17.7 ± 5.9 (neonatal—32)	20.7 ± 7.4 (10–48)	18.8 ± 6.6 (neonatal—48)
Seen at AMC (n, %)	50 (51%)	34 (62%)	84 (55%)
Genetic counseling ever (n, %)	12 (12%)	11 (20%)	33 (21%)
Genetic testing ever (n, %)	42 (42%)	25 (45%)	67 (44%)
**Patient-reported outcomes**			
IPQR consequences (dimension range 5–30)	21.2 ± 4.0 (10–30)	20.0 ± 5.1 (6–30)	20.8 ± 4.5 (6–30)
IPQR emotional representations (dimension range 5–30)	19.3 ± 5.7 (6–30)	17.8 ± 6.2 (6–30)	18.8 ± 5.9 (6–30)
IPQR illness coherence (dimension range 5–25)	18.2 ± 4.4 (6–25)	16.4 ± 4.7 (5–25)	17.6 ± 4.6 (5–25)
Zung SDS (dimension range 20–80)	43.5 ± 12.0 (20–70)	41.6 ± 11.4 (22–68)	42.8 ± 11.8 (20–70)

*AMC* academic medical center, *IPQR* Illness Perception Questionairre Revised, *SDS* self-rating depression scale

**Fig. 2:**
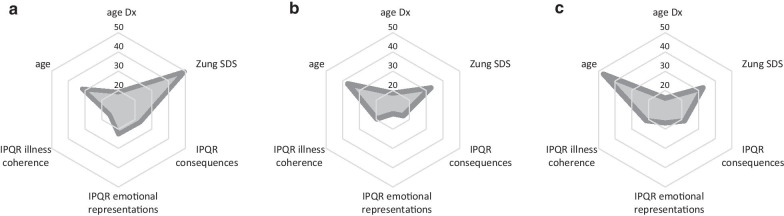
Three latent classes of patients with CHH/KS (n = 154). The LCMM analysis identified distinct subgroups based on demographic, clinical and patient-reported outcome data. **a** Class I (n = 84) was diagnosed significantly later (*p* = 0.045) and exhibits high SDS, disease consequences and emotional impact scores and low illness coherence (making sense of one’s disease). **b** Class II (n = 41) exhibited less severe psychosocial outcomes and greater illness coherence (all *p* < 0.001 vs. Class I). **c** Class III (n = 29) was diagnosed the earliest and exhibited relatively modest psychosocial impact. *Dx* diagnosis, *SDS* self-rating depression scale, *IPQR* Illness Perception Questionnaire-Revised

Accordingly, latent class analysis revealed the model with three subgroups demonstrated the best fit. The 154 subjects were classified as being a member of class I (n = 84 [54.5%]), class II (n = 41 [26.6%]) or Class III (n = 29 [18.8%]). We used maximum likelihood estimation with robust standard errors in an iterative process to determine parameters within the three classes and to generate probabilities of each participant belonging to each class. The classification probabilities for the most likely latent class membership (i.e. posterior probabilities) were acceptable (class I = 0.836, II = 0.906, III = 0.937, entropy = 0.80). Radar graphs of the three distinct profiles are shown in Fig. 2. Mean values with 95% confidence intervals for each continuous variable are shown in Table 2. In terms of the categorical variable education, high school education had a weak, negatively association with Class I membership (χ^2^ = − 0.575, *p* = 0.024) while having post-graduate education was strongly associated (χ^2^ = 4.392, *p* < 0.001). Having a college/university education was positively associated with membership in Class III (χ^2^ = 1.869, *p* = 0.028). Having been seen at a specialty/academic medical center was not significantly associated with membership in any of the classes.

**Table 2:** Mean values for continuous variables by class

Class	Age (years) (range 18–68)	Age at Dx (years) (range NN-48)	Illness Perception Questionnaire-Revised			Zung SDS (range 20–80)
			Consequences (range 5–30)	Emotional representations (range 5–30)	Illness coherence (range 5–25)	
	F = 36.78 *p* < 0.001	F = 3.17 *p* = 0.045	F = 52.26 *p* < 0.001	F = 112.5 *p* < 0.001	F = 38.96 *p* < 0.001	F = 51.35 *p* < 0.001
I (n = 84)	31.7 ± 8.2 (29.9–33.5)	19.2 ± 16.7 (17.8–20.7)	22.9 ± 3.5 (22.1–23.7)	22.7 ± 3.7 (21.9–23.5)	15.2 ± 4.0 (14.3–16.0)	49.6 ± 9.5 (47.6–51.7)
II (n = 41)	37.0 ± 7.6^†^ (34.6–39.4)	18.6 ± 4.9 (17.1–20.2)	16.2 ± 3.6^‡^ (15.0–17.3)	12.1 ± 3.7^‡^ (10.9–13.2)	19.6 ± 3.7^‡^ (18.4–20.7)	32.8 ± 9.9^‡^ (29.7–35.9)
III (n = 29)	47.0 ± 9.6^‡^ 43.4–50.6)	16.0 ± 5.3 * (13.9–18.0)	21.6 ± 3.1^‡^ (20.4–22.8)	16.9 ± 4.0^‡^ (15.4–18.5)	21.4 ± 2.6^‡^ (20.4–22.4)	32.7 ± 9.0^‡^ (32.7–39.5)

Among class differences depicted using F and *p* values; data are shown as mean ± SD (95% confidence interval); *Dx* diagnosis, *NN* neonatal, *SDS* self-rating depression scale; ANOVA with Sheffe post hoc test *p < 0.05 vs. Class I, ^†^*p* < 0.005 vs. class I; ^‡^*p* < 0.001 vs. class I

Compared to other classes, class I was diagnosed significantly later (Sheffe post hoc* p* < 0.05) and had significantly more IPQ-R consequences, greater IPQ-R emotional impact, and lower IPQ-R illness coherence (i.e. how one makes sense of their disease) (all *p* < 0.001) (Table 2). Class I also exhibited significantly higher Zung SDS scores (measuring depressive symptoms) than either of the other subgroups (Sheffe post hoc* p* < 0.001). Class II and III exhibit SDS scores in the normal reference range (i.e. 20–39) yet class I SDS scores (95% CI 47.6–51.7) fell squarely in the rage of moderate depressive symptoms (SDS range 48–55) akin to dysthymia or depression typically seen in the ambulatory setting [31]. These empirical data point to psychosocial sequelae associated with later diagnosis. As an exploratory step, we performed linear regression to identify predictors of older age at diagnosis among the patient reported outcome (i.e. IPQR consequences, IPQR emotional representations, IPQR illness coherence, Zung SDS). With the stepwise model selection procedures, only illness coherence was retained. Thus, the multivariate linear regression model is equivalent to a Pearson’s correlation. Illness coherence was negatively correlated with age at diagnosis (r = − 0.192, *p* = 0.009), consistent with a small-to-medium effect size (i.e. 0.1–0.3). Thus, older age at diagnosis is associated with making less sense of the illness (CHH/KS).

## Discussion

Herein we present findings of LCMM on a previously recruited cohort (n = 154) of patients with CHH/KS. The iterative Bayesian analytic approach identified three distinct subgroups (classes) of patients who were recruited online in collaboration with a patient support organization. These findings refute prior critiques of online community-based participatory methods for recruiting rare disease patients. Specifically, we identified three subgroups spanning a range of ages and psychosocial adaption (i.e. illness perceptions and depression symptoms). The present findings suggest this research methodology does not recruit a biased, homogenous sample with disproportionately higher needs than the general patient population. Prior work demonstrates that rare disease patients are internet “power-users” who frequently go online to seek information about their condition and find peer-to-peer support [5]. Indeed, a number of studies point to the important role the internet and social media has for patients and families living with rare diseases [17, 34–37]. Given the avid use of the internet by rare disease patients, researchers have utilized this avenue expand recruitment [6–8]. Moreover, the European Reference Network on Rare Endocrine Conditions (ENDO-ERN) highlights effective partnerships with patient organizations for conducting needs assessments [14, 17, 24, 25] and co-creating patient-facing materials [18, 19] as a model for facilitating clinic trials and improving clinical care for rare diseases [38].

As initially depicted in the 2011 landmark EURORDIS report [4], the so-called “diagnostic odyssey” is a common experience across rare diseases. Importantly, recent reports demonstrate the problem of delayed diagnosis has persisted in the field of rare diseases [39, 40]. Published literature on CHH/KS has suggested that later diagnosis is associated with poorer psychosocial outcomes (i.e. wellbeing and health-related quality of life) [12]. The cohort presented here is the largest group of prospectively recruited CHH/KS patients with measures relating to psychosocial outcomes (i.e. IPQ-R, SDS). The LCMM analysis of the CHH/KS cohort (n = 154) identified a subgroup (class I) was diagnosed significantly later (19.2 ± 6.7 years, 95% CI 17.8–20.7, *p* = 0.045) and had significantly worse patient-reported outcomes relating to psychosocial function (all *p* < 0.001). These data provide the first empirical evidence of the negative psychosocial sequelae related to later diagnosis. These data provide further impetus for increased attention to timely diagnosis [12, 15, 41]. Notably, the present findings provide new insights into coping and adaptation that were recently highlighted in a publication co-authored with a patient support group leader outlining a roadmap for supporting psychological adaptation related to CHH/KS [15].

Limitations of the present study include the limited sample size (n = 154). However, given the rarity of CHH/KS (i.e. 1:48,000 [13]), this cohort represents the largest prospectively recruited cohort in the literature. The LCMM findings demonstrate the utility of this analytic approach for identifying subgroups within cohorts despite limited sample size. Future directions may include broader application of LCMM to identify patient subgroups to inform tailored approached to treatment of rare diseases. Similarly, latent growth mixture modeling could be employed as a data-centered analytic strategy to identify distinct trajectories (i.e. natural history, treatment response) in rare disease populations [42].

## Conclusions

Rare disease research has traditionally been hampered by geographically dispersed patient populations resulting in studies with limited sample size and power. Partnerships with patient organizations combined with online data collection have emerged as approaches to overcome geographic roadblocks in rare disease research. Using LCMM, we counter critiques of such approaches. Rare disease patients recruited online in partnership with patient organizations are not a monolithic group. We identify three distinct latent subgroups (classes) spanning a spectrum of age, clinical experiences (age at diagnosis) and measures of coping (illness perceptions and depressive symptoms). These data support the validity of using community based participatory methods and online data collection for rare disease research. Moreover, we show the first empirical evidence that later age of CHH/KS diagnosis is associated with worse psychosocial outcomes. These findings underscore the importance of timely identification and initiation of treatment for improving health-related quality of life for patients with CHH/KS.

## Data Availability

De-identified data will be made readily available upon request for research purposes to qualified individuals within the scientific community.

## Abbreviations

AMC: Academic medical center
CHH: Congenital hypogonadotropic hypogonadism
COST: European Cooperation in Science and Technology
ENDO-ERN: European Reference Network on Rare Endocrine Conditions
EUCERD: European Union Committee of Experts on Rare Diseases
EURORDIS: European Organization for Rare Diseases
GnRH: Gonadotropin releasing hormone
KS: Kallmann syndrome
LCMM: Latent class mixture modeling
SD: Standard deviation
